# Could ^18^ F-DPA-714 PET imaging be interesting to use in the early post-stroke period?

**DOI:** 10.1186/s13550-014-0028-4

**Published:** 2014-06-06

**Authors:** Maria-Joao Ribeiro, Johnny Vercouillie, Severine Debiais, Jean-Philippe Cottier, Isabelle Bonnaud, Vincent Camus, Samuel Banister, Michael Kassiou, Nicolas Arlicot, Denis Guilloteau

**Affiliations:** 1 Université François Rabelais de Tours, Tours, UMR-S930, France; 2 Inserm U930, University of Tours, Tours 37000, France; 3 CHRU Tours, Tours 37000, France; 4 CIC-IT INSERM 806 Ultrasons et Radiopharmaceutiques, Tours, France; 5 CIC INSERM 202, Tours, France; 6 School of Chemistry, University of Sydney, Sydney 2006, New South Wales, Australia; 7 Brain and Mind Research Institute, Sydney 2050, New South Wales, Australia; 8 Discipline of Medical Radiation Sciences, University of Sydney, Sydney 2006, New South Wales, Australia; 9 Service de Médecine Nucléaire, Hôpital Bretonneau, 2, Boulevard Tonnellé, Tours CEDEX 37044, France

**Keywords:** Stroke, PET, 18 F-DPA-714, Neuroinflammation

## Abstract

**Background:**

Cerebral stroke is a severe and frequent condition that requires rapid and reliable diagnosis. If administered shortly after the first symptoms manifest themselves, IV thrombolysis has been shown to increase the functional prognosis by restoring brain reperfusion. However, a better understanding of the pathophysiology of stroke should help to identify potential new therapeutic targets. Stroke is known to induce an inflammatory brain reaction that involves overexpression of the 18-kDa translocator protein (TSPO) in glial cells and infiltrated leukocytes, which can be visualised by positron emission tomography (PET). We aimed to evaluate post-stroke neuroinflammation using the PET TSPO radioligand ^18^ F-DPA-714.

**Methods:**

Nine patients underwent ^18^ F-DPA-714 PET and magnetic resonance imaging (MRI) between 8 and 18 days after the ictus. Co-registration of MRI and PET images was used to define three volumes of interest (VOIs): core infarction, contralateral region, and cerebellum ipsilateral to the stroke lesion. Time activity curves were obtained from each VOI, and ratios of mean and maximum activities between the VOIs were calculated.

**Results:**

We observed an increased uptake of ^18^ F-DPA-714 co-localised with the infarct tissue and extension beyond the region corresponding to the damage in the blood brain barrier. No correlation was identified between ^18^ F-DPA-714 uptake and infarct volume. ^18^ F-DPA-714 uptake in ischemic lesion (mainly associated with TSPO expression in the infarct area and in the surrounding neighbourhood) slowly decreased from 10 min pi to the end of the PET acquisition, remaining higher than that in both contralateral region and ipsilateral cerebellum.

**Conclusion:**

Our results show that ^18^ F-DPA-714 uptake after acute ischemia is mainly associated with TSPO expression in the infarct area and in the surrounding neighbourhood. We also demonstrated that the kinetics of ^18^ F-DPA-714 differs in injured tissue compared to normal tissue. Therefore, ^18^ F-DPA-714 may be useful in assessing the extent of neuroinflammation associated with acute stroke and could also help to predict clinical outcomes and functional recovery, as well as to assess therapeutic strategies, such as the use of neuroprotective/anti-inflammatory drugs.

## Background

Cerebral stroke is a severe and frequently occurring condition that represents a leading cause of mortality and morbidity worldwide [1,2], being also the main aetiology of adult-acquired disabilities [3,4]. Stroke is haemorrhagic in 10% to 15% of cases but more often it is ischemic (85% to 90%) [3]. Due to arterial occlusion, ischemic stroke is a diagnostic and therapeutic priority; emergency treatment (e.g., intravenous thrombolysis) should be administered in the first hours after symptom onset. Unfortunately, no other emergency drug treatment has been validated, but an improved understanding of the pathophysiology of early cerebral ischemia should identify potential molecular targets, particularly for neuroprotective treatments. Therefore, promising neuroprotective drugs, which demonstrated effectiveness in animals, have been evaluated; however, these results remain unconfirmed in clinical trials [5,6]. Moreover, there is a lack of reliable imaging strategies to assess brain neuroinflammation after stroke [7-9].

Cerebral ischemia rapidly evolves to necrosis and a peri-necrosis area of ischemic penumbra, in which the brain tissue is still viable for a few hours. This cerebral tissue can be preserved if treatment is initiated quickly to restore the cerebral blood flow. Thus, this area of ‘darkness’ is the prime target for potential neuroprotective drugs. In animals, the infarcted area has been shown to expand in 24 h after the occlusion of a cerebral artery [10,11].

Although different mechanisms are involved in the pathogenesis of stroke, increasing evidence suggests that inflammation, mainly involving the microglial and the immune system cells, account for its pathogenic progression [11].

Experimental studies have demonstrated that microglial cells are the first inflammatory cells activated after the onset of cerebral ischemia [12]. After breakdown of the blood-brain barrier (BBB), which accompanies cerebral ischemia, the perivascular microglia and macrophages are activated; this activation appears to be at least partially responsible for the inflammatory lesions in cerebral infarctions [12]. Microglia, which constitute up to 10% of the total brain cell population, change from a resting to an activated state in response to central nervous system insults, and this change stimulates these cells for phagocytosis. Several studies focusing on the inflammatory reaction during the first days after stroke have demonstrated that this inflammatory response changes dramatically over time [13,14]. Therefore, the inflammatory markers correlated to the time course must be considered for any anti-inflammatory treatment approach in patients with acute ischemic strokes [14].

In humans, microglial activation can be assessed *in vivo* through neuroimaging of the 18-kDa translocator protein (TSPO) with selective TSPO radioligands. TSPO, formerly known as peripheral benzodiazepine receptor (PBR), is part of a multimeric ‘protein complex’ associated with the outer mitochondrial membrane of many cells [15]. TSPO is present in peripheral tissues and also in glial cells, but in the healthy brain, its expression is minimal [16]. TSPO may therefore be a valuable biomarker of inflammation, as it is highly expressed in phagocytic inflammatory cells.

A large number of positron emission tomography (PET) and single photon emission computerised tomography (SPECT) radioligands, selective for TSPO, have been developed, of which ^11^C-PK11195 was the first to be evaluated [17]. Studies using ^11^C-PK11195 demonstrated increased binding of this radiotracer around the outer border of ischemic lesions several days after stroke, as well as in distant areas from the lesion [18-23]. This increased uptake was observed from 3 days after ictus, reaching its maximum at 7 days, and continuing for a period of 4 weeks [9].

However, the 20-min radioactive half-life of ^11^C-PK11195 is a serious drawback to the increased accessibility of biomarkers for routine clinical purposes since the use of these markers is limited to centres with an on-site cyclotron. Fluorine-18-labelled ligands, therefore, appear to be the best alternative, as the 110-min half-life of fluorine-18 enables centralised production and loco-regional delivery. Several groups have also evaluated neuroinflammation models in rats using the TSPO radioligand ^18^ F-DPA-714, concluding that it provides accurate quantitative information of TSPO density after cerebral ischemia, herpes encephalitis, amyotrophic lateral sclerosis, and gliomas [24-27]. Several studies have already demonstrated that the regional distribution of ^18^ F-DPA-714 aligned well with other PET studies using TSPO radioligands in the human brain [25,26].

The aim of the present study was to assess microglial activation after a recent stroke using ^18^ F-DPA-714, and to evaluate the relationship between ^18^ F-DPA-714 uptake and the infarct volume, also analysed by magnetic resonance imaging (MRI).

## Methods

### Subjects

This study was approved by the local Medical Bioethics Committee and was conducted according to French legislation and European directives.

Nine patients (Table 1) with recent unilateral cerebral infarcts were included. All patients underwent MRI and PET brain scans, with a maximum interval of 24 h between the two acquisitions. The following inclusion criteria were applied: Patients aged between 18 and 85 years, with a recent cerebral infarct involving the medial cerebral artery, visualised on computed tomography (CT) or MRI images, 6 to 20 days before the PET examination. None of the patients received the thrombolytic treatment, and none showed any symptoms suggesting the presence of any other significant neurodegenerative or psychiatric disease or had severe renal insufficiency, contraindicating the injection of a gadolinium chelate. No subject had been treated with anti-inflammatory drugs in the month preceding the PET study or with drugs that might interfere with ^18^ F-DPA-714 binding. All patients provided informed written consent before participating in the study.

**Table 1:** Clinical characteristics of the patients included in the study

**Patient**	**Gender**	**Injury vascular region**	**Age (years)**	**Weight (kg)**	**Time between stroke and PET (days)**
1	F	Right MCA	82	73	14
2	M	Left MCA	55	88	11
3	F	Right MCA	71	94	13
4	F	Left MCA	82	62	13
5	M	Right MCA	71	68	12
6	M	Right MCA	63	97	8
7	F	Right MCA	77	53	11
8	M	Left MCA	56	54	13
9	F	Right MCA	58	62	18

MCA, middle cerebral artery.

### Radiosynthesis

*N*,*N*-diethyl-2-(2-(4-(2-fluoroethoxy)phenyl)-5,7-dimethyl-pyrazolo[1,5-α]pyrimidin-3-yl)acetamide (DPA-714) was labelled with fluorine-18 at its 2-fluoroethyl moiety, following nucleophilic substitution of the corresponding tosylate analogue, according to slight modifications of previously reported procedures [28]. The formulation of ^18^ F-DPA-714 provided a sterile injectable solution of isotonic sodium chloride with ethanol at a mass percentage of <8% for total injected volumes (ranging from 3 to 5 mL), in accordance with the European Pharmacopoeia. The mean specific activity of ^18^ F-DPA-714 obtained was 113.9 ± 36.9 GBq/μmol.

### Imaging data acquisition

Brain MRI studies were performed using a 1.5 T imager (GE Healthcare, Milwaukee, WI, USA). Diffusion, FLAIR T2, T2*-weighted GRE sequences and high-resolution T1-weighted 3D MRI volumes were acquired for all subjects before and after the gadolinium administration (Gadobenate dimeglumine, Bracco imaging®, Ceriano Laghetto, Italy).

FLAIR T2-weighted and diffusion-weighted images showed the extent of cerebral infarction, whereas the T1-weighted SPGR image was used to allow co-registration with the PET images. After the gadolinium injection, axial volumetric T1 acquisition was performed to clarify the extent of the BBB breakdown.

PET studies were performed using a Dual Gemini (Philips Medical Systems, Amsterdam, the Netherlands), a whole-body hybrid PET/CT scanner with a full-width-half-maximum (FWHM) resolution of 5 mm in all directions, in 3D acquisition mode. To perform attenuation correction, a low dose CT helical scan was done (scan field, 600 mm; increments, 5 mm; slice thickness, 3.2 mm; pitch, 1.5, 0.75 second per rotation; 512 × 512 matrix; 120 kV; 80 mAs). Acquisition data were reconstructed with the standard package included with the system (PET view software-Philips Medical Systems). PET sinograms were corrected for tissue attenuation, decay, scatter, and random radiation, and then they were reconstructed using a 3D iterative RAMLA algorithm in voxels of 2 × 2 × 2 mm^3^.

To limit incidental movement, the patient's head was positioned in a headrest using a 3D laser alignment. All cerebral PET examinations were acquired in list mode over 90 min, after IV injection of 244.0 ± 27.4 MBq of ^18^ F-DPA-714.

### Data analysis

An integrated PET image was obtained for each subject from the 90-min acquisition. This image was used to perform co-registration with each corresponding MRI image (T1-weighted SPGR and post-contrast images). Non-rigid registration was performed using normalised mutual information and PMOD® 3.4 software.

For each subject, a volume of interest (VOI) was defined over the injured tissue (IT) using individual MRI images. A similar VOI was transposed according to the region on the contralateral (CL) unaffected hemisphere. A VOI was also defined over the ipsilateral cerebellum to the injured tissue. All of VOIs were transposed onto PET images to obtain time activity curves (TACs). TACs were used to evaluate the kinetics of ^18^ F-DPA-714 over the 90-min acquisition. Ratios between the mean activity of the injured tissue VOIs and the contralateral (IT/CL) and cerebellar (IT/C) VOIs were calculated. We also determined similar ratios using the maximal activity measured in the same VOI.

For the nine patients, the infarct volumes (cm^3^) were calculated using the corresponding MRI images. Infarct volumes were manually outlined on diffusion-weighted MR images by an experienced neuroradiologist using an imaging analysis tool (functool, advantage windows, version 4.3; GE healthcare).

### Statistical analysis

All data are presented as the mean ± standard deviation of the mean (SD). Significance was set at p < 0.05. Wilcoxon signed-rank test for paired values was used to determine the significant difference between VOIs ratios. Spearman's correlation test was used to assess the relationship between the volume of the lesion and ^18^ F-DPA-714 uptake ratios and between lesion volume measured by MRI and PET.

## Results

The patients' clinical characteristics are summarised in Table 1. No adverse or subjective effects were observed for any of the nine subjects studied after injecting an average of 244.0 ± 27.4 MBq of ^18^ F-DPA-714. The imaging studies were performed 12.6 ± 2.7 days (min 8 days, max 18 days) after the acute injury.

**Figure 1:**
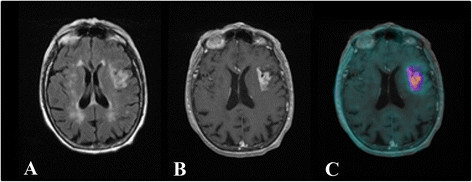
**Axial images obtained after vascular injury.** The imaging studies were performed 13 days post-stroke. The infarct volume evaluated on MRI was 13 cm^3^. **(A)** Flair T2-weighted MR image. Left fronto-insular hypersignal demonstrating infarcted tissue. **(B)** Post-gadolinium T1-weighted MR image. Enhancement signal in the left fronto-insular cortex corresponding to the breakdown of BBB. **(C)** Co-registration image obtained between the post-gadolinium T1 MRI and PET. Note the absence of ^18^ F-DPA-714 uptake by the multiple bilateral nodular hypersignals within the white matter in connection with leukoaraïosis lesions.

Figure 1 shows an example of MRI and ^18^ F-DPA-714 images obtained from one patient (patient 4). For this patient, we observed that the radiopharmaceutical uptake is relatively comparable to injury tissue highlighted by MRI.

**Figure 2:**
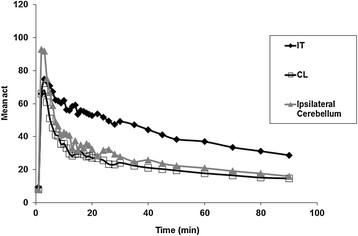
**TACs of**^**18**^ **F-DPA-714 binding on the injured tissue (IT), contralateral (CL) normal tissue and ipsilateral cerebellum.**

**Figure 3:**
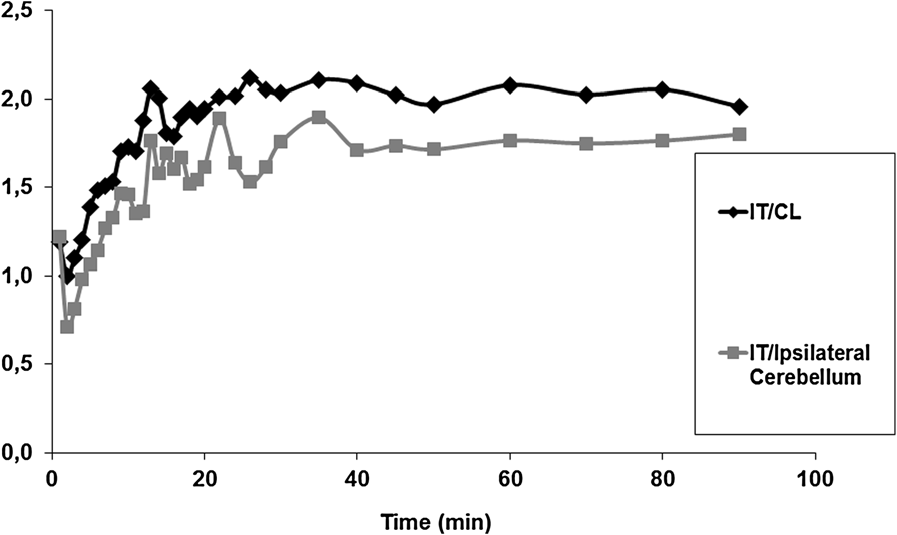
Ratio values calculated between injured tissue and two reference regions (contralateral (CL) and ipsilateral cerebellum).

Figure 2 depicts a representative TAC of ^18^ F-DPA-714 uptake in the injured tissue and in the intact counterpart contralateral region and ipsilateral cerebellum (both used as reference regions) from a single subject. Figure 3 shows the evolution of the ratios calculated between the mean activity of the injured region and the two reference regions for the same subject. For all patients, the TAC revealed an initial rapid accumulation of radioactivity in the injured tissue, with a peak observed at 5 min post-injection (pi), followed by a gradual cerebral clearance from 10 to 20 min pi to the end of the PET acquisition. For the two reference regions, lower binding values of ^18^ F-DPA-714 were observed, with a maximum uptake at 5 min pi followed by two decreased phases from 5 to 20 min pi and from 10 to 20 min pi to the end of the acquisition. Moreover, the binding of ^18^ F-DPA-714 was lower for the contralateral region than for the cerebellum. For all subjects, the ^18^ F-DPA-714 uptake in the infarcted tissue remained higher than that observed for the other two regions during the entire acquisition.

Using the two reference regions at 40 and 80 min pi, the ratios obtained for the nine patients are presented in Table 2. The ratios using the mean activities were higher, although they were significantly different only at 40 min pi, when the contralateral VOI was used as the reference region, compared with the ratios using the cerebellum (IT/CL = 1.78 ± 0.31 and IT/Cerebellum = 1.55 ± 0.21 at 40 min, *p* = 0.020; IT/CL = 1.99 ± 0.56 and IT/Cerebellum = 1.79 ± 0.40 at 80 min pi, *p* = 0.155 respectively). Using the maximal activity calculated for each VOI, the ratios were also higher, however not significantly different, when using contralateral VOI (IT/CL = 2.05 ± 0.54 and IT/Cerebellum = 2.02 ± 0.64 at 40 min, *p* = 0.911; IT/CL = 2.44 ± 0.72 and IT/Cerebellum = 2.19 ± 0.54 at 80 min pi, *p* = 0.164, respectively).

**Table 2:** **Volumes (cm**^**3**^**) of infarcted region and VOIs and individual ratio values**

**Patient**	**Infarct volume**	**VOI volume**	**40 min post-injection**				**80 min post-injection**			
	**(cm**^**3**^**)**	**(cm**^**3**^**)**	**IT/CL**		**IT/Ipsilateral cerebellum**		**IT/CL**		**IT/Ipsilateral cerebellum**	
	**MRI**	**PET**	**Mean**	**Max**	**Mean**	**Max**	**Mean**	**Max**	**Mean**	**Max**
1	108	93.0	2.03	2.75	1.70	2.70	2.03	2.85	1.77	2.47
2	16	6.0	1.14	1.08	1.26	1.21	1.28	1.27	1.29	1.30
3	190	163.8	2.09	2.11	159	1.82	3.27	3.42	2.55	2.59
4	13.1	17.3	2.10	2.61	1.80	2.89	2.05	2.32	1.77	2.36
5	34.6	32.3	1.93	2.14	1.55	2.54	2.08	3.41	1.64	2.62
6	3.4	3.5	1.54	1.84	1.17	1.47	1.46	1.67	1.25	1.39
7	0.7	0.73	1.83	1.68	1.73	2.52	2.08	2.39	1.82	2.82
8	12.9	13.7	1.73	2.54	1.67	1.60	1.69	2.52	2.07	2.23
9	1.6	1.8	1.66	1.67	1.48	1.42	1.95	2.15	1.96	1.89
Mean			1.78	2.05	1.55	2.02	1.99	2.44	1.79	2.19
SD			0.31	0.54	0.21	0.64	0.56	0.72	0.40	0.54

For each VOI, mean activity and maximal (Max) activity were used. The mean and standard deviation (SD) for these ratios are also indicated. Individual ratio values were calculated between the injured tissue (IT, necrotic and inflammatory) and the two reference regions, contralateral (CL) and ipsilateral cerebellum.

The median lesion volume estimated by MRI was 13.1 cm^3^ [range 0.7 (subject 7) to 190 cm^3^ (subject 3)]. There was no statistically significant relationship between this volume and the ^18^ F-DPA-714 uptake, according to Spearman's test (*p* = 0.24). However, a strong correlation was observed between the lesion volume estimated by MRI and the VOI volume used to calculate the activity concentration (*r* = 0.95 end *p* = 0.0003, Spearman's test).

## Discussion

In this study, all imaging scans were performed within 12.6 ± 2.7 days after the ictus. Studies performed with the reference TSPO radioligand ^11^C-PK11195 in stroke patients indicated that the binding became significant a few days after the stroke onset, subsequently increased in approximately 1 week, and declined after 3 to 4 weeks [18,19,29]. A more recent study with ^11^C-PK11195, conducted 2 to 3 weeks post-stroke in a group of 18 subjects confirmed the local activation of microglia with increased radioligand uptake in the infarcted area [23]. Moreover, activation in the peri-infarct area appears to be more intense than in the core [30]. The value of our study is that it demonstrates the ability of a TSPO fluorine radioligand to map inflammatory process, in both the infarct and the surrounding surviving tissues. Indeed, one of the main difficulties in validating new radiopharmaceuticals as biomarkers of neuroinflammation lies in ruling out the possibility of non-specific binding, particularly passive leakage through a damaged BBB, as well as potential infiltration of peripheral circulating monocytes/macrophages within the brain parenchyma.

We observed that the increased uptake of ^18^ F-DPA-714 on PET images differed, even if very moderately, from the MRI gadolinium-enhanced regions, and was not limited to the infarct tissue with BBB breakdown; it also included non-infarcted cerebral tissue. This finding most likely reflects the activation of microglia with an increase in TSPO expression at the periphery of the necrotic lesion. On the other hand, we cannot forget that the PET spatial resolution is lower than that of the MRI's.

Moreover, the radiotracer uptake was not correlated with the infarct volume measured by MRI. This point is in accordance with a previous USPIO MRI study performed in subacute stroke patients demonstrating that the extent of the inflammatory response after stroke was unrelated to the lesion volume [31].

To our knowledge, only one study has compared two different TSPO radioligands in post-stroke patients (i.e., vinpocetine and PK11195, both labelled with carbon-11) [32]. The authors compared the diagnostic potential of these radioligands to visualise activated microglia in four post-stroke patients. They observed that the binding potential for ^11^C-vinpocetine was greater than that obtained for ^11^C-PK11195 in all evaluated regions, but the differences did not reach significance. ^11^C-vinpocetine binding potential was greater in the peri-infarcted zone than in the ischaemic core, but the difference did not prove to be significant [32,33].

However, a direct comparison between ^18^ F-DPA-714 and ^11^C-PK11195 labelling, on a model of cerebral ischemia in rats, was done by Boutin et al. [34]. ^18^ F-DPA-714 displayed a higher signal-to-noise ratio than ^11^C-PK11195, suggesting that with the longer half-life of fluorine-18, ^18^ F-DPA-714 could be a good alternative for TSPO imaging.

The TACs generated for each subject showed that ^18^ F-DPA-714 uptake in the lesion, as well as in the contralateral cerebral tissue and the cerebellum, reached peak values at 5 min pi. The TACs for the injured region showed a slow decrease in binding from the maximum uptake to the end of the acquisition. However, this kinetic analysis showed two decreased phases for the other two regions: (1) a faster phase, between 5 and approximately 20 min, and (2) a slower phase, from 20 min pi to the end of the acquisition. This evolution of the kinetics of ^18^ F-DPA-714 in the cerebellum and in the normal cerebral tissue aligned with that observed in a previous study performed in a group of seven healthy volunteers [25]. Indeed, in this previous study, we observed that the kinetics of the radioligand was similar for all cortical regions and the cerebellum.

On the other hand, stroke patients may show remote metabolic changes in the cerebellum resulting from diaschisis [25,35]. Although microglial changes, as a consequence of cerebellar diaschisis, are not known, we have chosen to take into account only the cerebellar hemisphere ipsilateral to the stroke to define the reference region.

The evolution of the ^18^ F-DPA-714 uptake in humans differed from that reported by Martin et al. in an ischemia model in rats [36]. The authors showed that the ^18^ F-DPA-714 uptake in the lesion reached a peak value at 30 min and remained stable until the end of the acquisition. In our study, we observed an earlier peak at 5 min pi followed by a slower decrease.

We also demonstrated that the binding ratio of ^18^ F-DPA-714 was higher when the contralateral VOI was used as a reference region, compared to when the cerebellum was used as a reference region. Several authors have previously documented the fact that the cerebellum in humans was not devoid of TSPO [37] and it would be of interest in future TSPO PET imaging studies to focus on focal inflammation, including stroke, Rasmussen encephalitis, epilepsy, and traumatic brain injury, and to consider the contralateral area as a suitable and TSPO-expression free reference tissue. Nevertheless, Martin et al. also detected increased ^18^ F-DPA-714 binding in the contralateral side at 11 days after focal cerebral ischemia in rats. They concluded that this finding was probably due to different mechanisms related to the expansion of infarction, such as spreading depression [36]. Thus, the exact fraction of specific binding to TSPO, in both the contralateral region and the cerebellum, would have to be determined by a blocking study requiring substantial doses of a blocking agent, which was ethically inconsistent with the present study. Interestingly, the ratio values calculated in the present study between the injured tissue and the contralateral region at 40 min post-^18^ F-DPA-714 injection, using the mean activity values, were significantly higher (*p* = 0.020, Mann-Whitney *U* test) than those previously published by Gerhard et al. using ^11^C-PK11195, obtained with three acquisitions from patients with the same specifications (interval in the range of 6 to 20 days between stroke ictus and PET acquisition) [18]. Although this comparison of two separate studies should be considered cautiously, it suggests that ^18^ F-DPA-714 might represent a good fluorine-18 alternative to ^11^C-PK11195 for TSPO PET imaging, with the higher ratio of ipsilateral-to-contralateral uptake. Moreover, our results are in accordance with preclinical direct comparison of both radiopharmaceuticals in a rodent model of focal ischemia [34], demonstrating that ^18^ F-DPA-714 achieved *in vivo* a higher ratio of ipsilateral uptake to contralateral uptake than ^11^C-PK11195.

Several studies have demonstrated the existence of different binder populations for ^11^C-PBR28, for which approximately 10% of the population appeared to be non-binders [38]. Owen and colleagues evidenced 3 types of binding pattern: high-affinity binders (approximately 50%), low-affinity binders (approximately 20%) and mixed-affinity binders (approximately 30%), related to a single-nucleotide polymorphism (rs6971) within the human TSPO gene [39], and they extended this finding to other PET TSPO radioligands, including the carbon 11-radiolabelled derivative of ^18^ F-DPA-714, namely, DPA-713 [40]. One limitation of our study is that we did not directly identify the subjects with low or high affinity for TSPO. It is likely that ^18^ F-DPA-714 is also sensitive to this variable inter-individual affinity state. Nevertheless, all patients in our study demonstrated increased ^18^ F-DPA-714 uptake by the injured tissue.

## Conclusions

Our results show that the uptake of ^18^ F-DPA-714 occurred not only due to the BBB breakdown, but also because of the activation of microglia in the area surrounding the infarct. We also showed that the kinetics of ^18^ F-DPA-714 differ between injured and normal tissues. The ability to assess microglial activation *in vivo* may improve our understanding of the mechanisms of neuroinflammation in acute disorders such as stroke, and should enable effective treatment monitoring. This study also demonstrates that a 10 to 20 min of PET emission acquisition, performed between 40 and 80 min pi, was able to differentiate acute tissue injury from normal brain tissue.

## Competing interests

All authors confirm that they have no applicable financial disclosures and no current or potential conflicts of interest.

## Authors' contributions

MJR and JPC performed data acquisition and analysis. SB and MK performed the precursor production. JV, NA, and DG performed the radiosynthesis and pharmacological controls. SD and IB have recruited the patients and performed clinical examinations. NA performed statistical analysis. MJR, JV, JPC, VC, NA and DG drafted the manuscript. MJR, JPC, SD, IB, and DG conceived the study and participated in its design and coordination. All authors read and approved the final manuscript.
